# Characterizing *PALB2* intragenic duplication breakpoints in a triple-negative breast cancer case using long-read sequencing

**DOI:** 10.3389/fonc.2024.1355715

**Published:** 2024-02-28

**Authors:** Iulian O. Ban, Alice Chabert, Thomas Guignard, Jacques Puechberty, Simon Cabello-Aguilar, Pascal Pujol, Julie A. Vendrell, Jérôme Solassol

**Affiliations:** ^1^ Laboratoire de Biologie des Tumeurs Solides, Département de Pathologie et Oncobiologie, Centre Hospitalier Universitaire (CHU) Montpellier, Université de Montpellier, Montpellier, France; ^2^ Department of Medical Genetics, Arnaud de Villeneuve Hospital, Montpellier, France; ^3^ Laboratoire de Génétique Chromosomique, Plateforme ChromoStem, Centre Hospitalier Universitaire (CHU) de Montpellier, Université de Montpellier, Montpellier, France; ^4^ Montpellier BioInformatics for Clinical Diagnosis (MOBIDIC), Molecular Medicine and Genomics Platform (PMMG), Centre Hospitalier Universitaire (CHU) Montpellier, Montpellier, France; ^5^ Institut de Recherche en Cancérologie de Montpellier (IRCM), Univ Montpellier, Inserm, Institut du Cancer de Montpellier (ICM), Montpellier, France

**Keywords:** PALB2, LGR, breast cancer, long-read sequencing, molecular alteration

## Abstract

**Introduction:**

Accurate identification and characterization of Large Genomic Rearrangements (LGR), especially duplications, are crucial for precise diagnosis and risk assessment. In this report, we characterized an intragenic duplication breakpoint of *PALB2* to determine its pathogenicity significance.

**Methods:**

A 52-year-old female with triple-negative breast cancer was diagnosed with a novel *PALB2* LGR. An efficient and accurate methodology was applied, combining long-read sequencing and transcript analysis for the rapid characterization of the duplication.

**Results:**

Duplication of exons 5 and 6 of *PALB2* was validated by transcript analysis. Long-read sequencing enabled the localization of breakpoints within *Alu* elements, providing insights into the mechanism of duplication via non-allelic homologous recombination.

**Conclusion:**

Using our combined methodology, we reclassified the *PALB2* duplication as a pathogenic variant. This reclassification suggests a possible causative link between this specific genetic alteration and the aggressive phenotype of the patient.

## 1 Introduction

Hereditary Breast and Ovarian Cancer (HBOC) syndrome predominantly arises from pathogenic alteration within the *BRCA1* and *BRCA2* genes (1). However, genes, such as *PALB2*, *RAD51D* and *RAD51C* have gained recognition as susceptibility markers and are routinely included in HBOC diagnostic screenings (among others) (2, 3). Characterized as a tumor suppressor, PALB2 (Partner and Localizer of BRCA2) plays an essential role in maintaining genomic integrity through its involvement in homologous recombination repair (HRR) pathway (4). Central to this process, it recruits BRCA2 and RAD51 to DNA lesions and amplifies strand-invasion function of RAD51, all while facilitating the formation of the integral BRCA1-PALB2-BRCA2 complex (5). Extensive research has established that pathogenic alterations in *PALB2* are associated with a substantially increased risk, up to seven-fold, for developing breast cancer and other related cancers (6, 7). About 70% of breast tumors in individuals carrying *PALB2* variations are estrogen receptor-positive, similar to patterns observed in patients with *BRCA2* genetic alterations and sporadic breast cancer. Notably, among individuals with *PALB2* variants, about 30% of breast tumors exhibit a triple-negative profile, which surpasses the typical range of 12%-17% observed in general breast cancer (8). Thereby, individuals who are heterozygous for germline loss-of-function variants in *PALB2* often present more aggressive clinical and pathological features, including the triple-negative breast cancer phenotype and a younger age at diagnosis (9, 10).

In the field of diagnosis, molecular characterization of variants has predominantly focused on single nucleotide substitutions (SNV) and small insertions/deletions. Additionally, a more limited proportion of pathogenic alterations is attributed to large genomic rearrangements (LGR) (11). These variations are characterized by genomic segments that deviate from the typical two-copy diploid state, underscoring the genetic complexity inherent in numerous cancer disorders (12). Large genomic deletions within gene coding regions may emerge as pathogenic, notably if they disrupt the reading frame and lead to the production of non-functional proteins. However, duplications introduce a layer of complexity, making their pathogenicity difficult to determine (13). Current techniques, such as target capture next-generation sequencing (NGS) and multiplex ligation-dependent probe amplification (MLPA), are standard for assessing copy number variation CNVs. Nevertheless, these techniques cannot determine whether the duplications are in tandem or have translocated elsewhere in the genome. As a result, these LGR are commonly classified as variants of uncertain significance (VUS) (14). Additionally, the inherent uncertainty associated with the reporting of VUS presents a considerable obstacle for clinicians in providing conclusive medical guidance. This uncertainty not only affects clinical decision-making but also place patients in a state of psychological distress. Therefore, it is essential to broaden our characterization of these alterations to enhance clinical outcomes for patients.

Here, we identified a novel *PALB2* LGR, resulting in the duplication of exons 5 and 6 in a patient diagnosed with triple-negative breast cancer. An efficient and accurate methodology was applied, combining various methods for the rapid characterization and reclassification of the LGR, enabling the precise identification of duplication breakpoints.

## 2 Materials and methods

### 2.1 Patient

A proband 52-year-old female was referred to the laboratory for hereditary breast and ovarian cancer (HBOC) testing after the diagnosis of triple-negative bifocal invasive ductal adenocarcinoma of the left breast. Written informed consent was obtained from the patient after genetic counseling.

### 2.2 DNA extraction and NGS analysis

Genomic DNA (gDNA) was isolated from peripheral blood samples of the patient using the QiaSymphony^®^ DSP, DNA Midi kit (Qiagen, Hilden, Germany) according to the manufacturer’s recommendations. Extracted DNA was quantified using the Qubit dsDNA Broad Range Assay Kit in combination with a Qubit fluorimeter (Thermo Fisher Scientific, Waltham, MA, USA). Gene-targeted enrichment was performed with the SureSelectXT reagent kit with custom target enrichment probes (Agilent Technologies, Santa Clara, CA, USA) according to the manufacturer’s recommendations. The HBOC gene panel explored 13 genes (*MLH1, MSH2, EPCAM, MSH6, PMS2, PTEN, CDH1, BRCA1, BRCA2, PALB2, RAD51C, RAD51D, TP53*). Massively parallel sequencing was performed using the NextSeq 500 instrument (Illumina, San Diego, CA), with an average read depth of 1,000×. Bioinformatics analysis was conducted using the CE-IVD GermlineVar and CNVCapture pipelines (SeqOne Genomics, Montpellier, France).

### 2.3 Multiplex ligation-dependent probe amplification (MLPA)

Confirmation screening of *PALB2* LGR was performed on the gDNA from two independent blood samples employing the SALSA MLPA P260 *PALB2-RAD50-RAD51C-RAD51D* Probe mix (MRC Holland, Amsterdam, The Netherlands), following the supplier’s instructions. Amplicons were run on an ABI 3500XL (Applied Biosystems, Foster City, USA), and data were analyzed using Coffalyser.Net software (MRC Holland).

### 2.4 RNA analysis

RNA was isolated from peripheral blood utilizing PAXgene® blood RNA tubes (BD Biosciences) followed by extraction using the Maxwell^®^ RSC simplyRNA Blood Kit with DNase treatment (Promega, Madison, WI, USA) according to the manufacturer’s recommendations. Extracted RNA was quantified using the Qubit RNA HS Assay Kit (Thermo Fisher Scientific, Waltham, MA, USA). Synthesis of complementary DNA (cDNA) was performed using SuperScript II Reverse Transcriptase, random primers and RNaseOUT (Thermo Fisher Scientific Waltham, MA, USA). cDNA was amplified using primers specifically designed to selectively amplify the proband duplicated region (forward:5′-GAACACCTCCACCCATTGAG-3′; reverse:5′-TTGACTCAAAGGGCTCCACT-3′) and further sequenced on an ABI 3500XL (Applied Biosystems) using BigDye Terminator Chemistry (Thermo Fisher Scientific) according to the manufacturers’ recommendations. The results were analyzed with the Seqscape 4 and SeqA7 softwares (Applied Biosystems) using *PALB2* transcript reference NM_024675.3.

### 2.5 Long-read sequencing and breakpoint determination

Long-Range PCR amplification was performed using LongAmp® Hot Start Taq 2X Master Mix (New England Biolabs^®^) with specific primers flanking *PALB2* exon 4 to exon 7 (forward: 5′-GCAGAAAAACATTCTTGCACAG-3′; reverse: 5′-CAAAACATGGCACTCACATCT-3′) as described by the manufacturer’s instructions. Long-Range PCR products were purified using NucleoSpin Gel and PCR Clean-up (Macherey-Nagel, Düren, Germany). Library preparation was performed using the SQK-LSK114 kit (Oxford Nanopore Technologies, New York, NY, USA) according to the supplier’s protocol, and sequenced on a Flongle flow cell FLO-FLG114 MinION Mk1B device using MinKNOW software (23.04.5 version) for 18 hours, achieving a read depth of 1700X. Base-calling of the FAST5 files was performed using Guppy software (6.5.7) using high accuracy model (template r10.4.1_e8.2 400bps_hac.jsn). The resulting reads were aligned to the GRCh37 human reference genome using Minimap2 (2.26-r1175) and visualized using the Integrative Genomics Viewer (IGV). Reads containing the specified insertion motif were extracted using in-house scripts. Structural variant (SV) calling was subsequently executed using Sniffles to validate the presence of the duplication. Data were validated after amplification and sequencing on an ABI 3500XL using the following primers (forward: 5′-TGCCTCTCCTACTCAAATGGTG-3′; reverse: 5′-TTTCAAACTACTGGGC-3′). The Repeat Masker program was employed to identify *Alu* sequences and sequence alignment was performed using ClustalW software.

## 3 Results

### 3.1 Patient description and LGR detection

**Figure 1:**
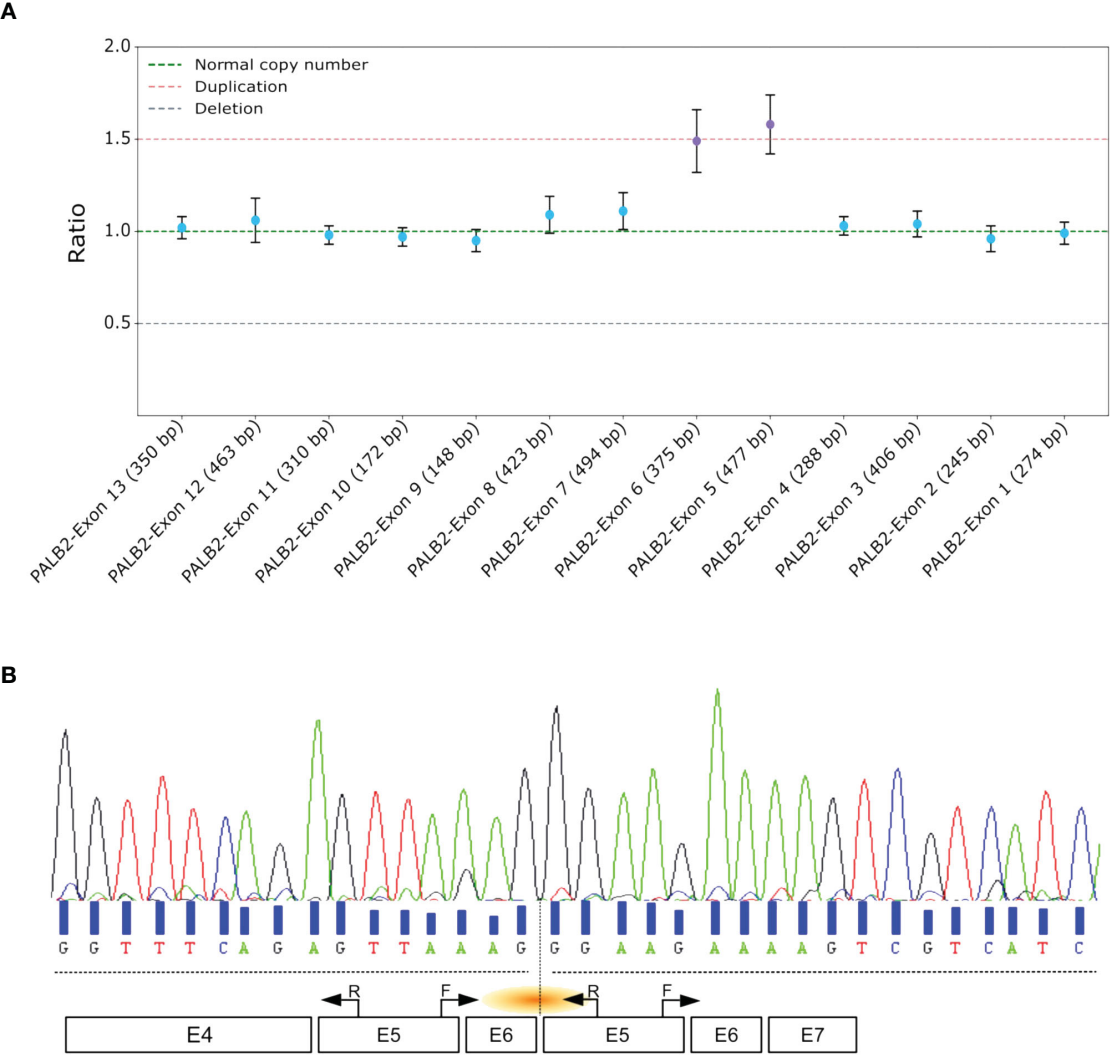
Comprehensive Analysis and Characterization of Tandem Duplications in *PALB2*. **(A)** MLPA assay of *PALB2*. Each dot represents the amplification ratio of a specific probe pair, reflecting the relative amount of the targeted DNA segment compared to three reference samples. Error bars indicate the standard deviation of the measured ratio. The ratio is calculated for each probe and indicates whether there is a deletion (ratio <1), duplication (ratio >1), or no change (ratio ≈1). **(B)** Sanger sequencing electropherogram of the proband’s cDNA, with black arrows marking the primer positions. The orange shallow points the targeted region. F, forward; R, reverse.

A 52-year-old woman was referred to our laboratory after being diagnosed with a stage III triple-negative bifocal invasive ductal carcinoma in the left breast. The patient’s family had no history of hereditary breast or ovarian cancer correct in cancers (**Supplementary Figure 1**). NGS identified a heterozygous germline duplication at the genomic level, encompassing exons 5 and 6 of *PALB2*. This finding was subsequently validated using MLPA (**Figure 1A**). Notably, no other pathogenic or likely pathogenic alterations were reported in the other genes explored in NGS HBOC-associated panel.

### 3.2 Transcript analysis

To validate the presence of the tandem duplication of exons 5 and 6 at the transcriptomic level, we designed primers that selectively amplified the region of interest from the cDNA. The forward primer was positioned at the end of exon 5, and the reverse primer at the start of the same exon. This approach specifically targeted an amplification product spanning exons 5-6-5 (**Figure 1B**). A distinct 361 base pair PCR amplification was evident in the proband sample, whereas no amplification was detected in the control sample. Sanger sequencing of the amplified cDNA confirmed the tandem nature of this duplication (**Figure 1B**). Furthermore, this aberrant transcript induced a frameshift, the Asparagine at position 863 was replaced by Glycine, leading to the appearance of a stop codon 37 amino acids downstream p.(Asn863Glyfs*37).

### 3.3 Genomic breakpoint determination

**Figure 2:**
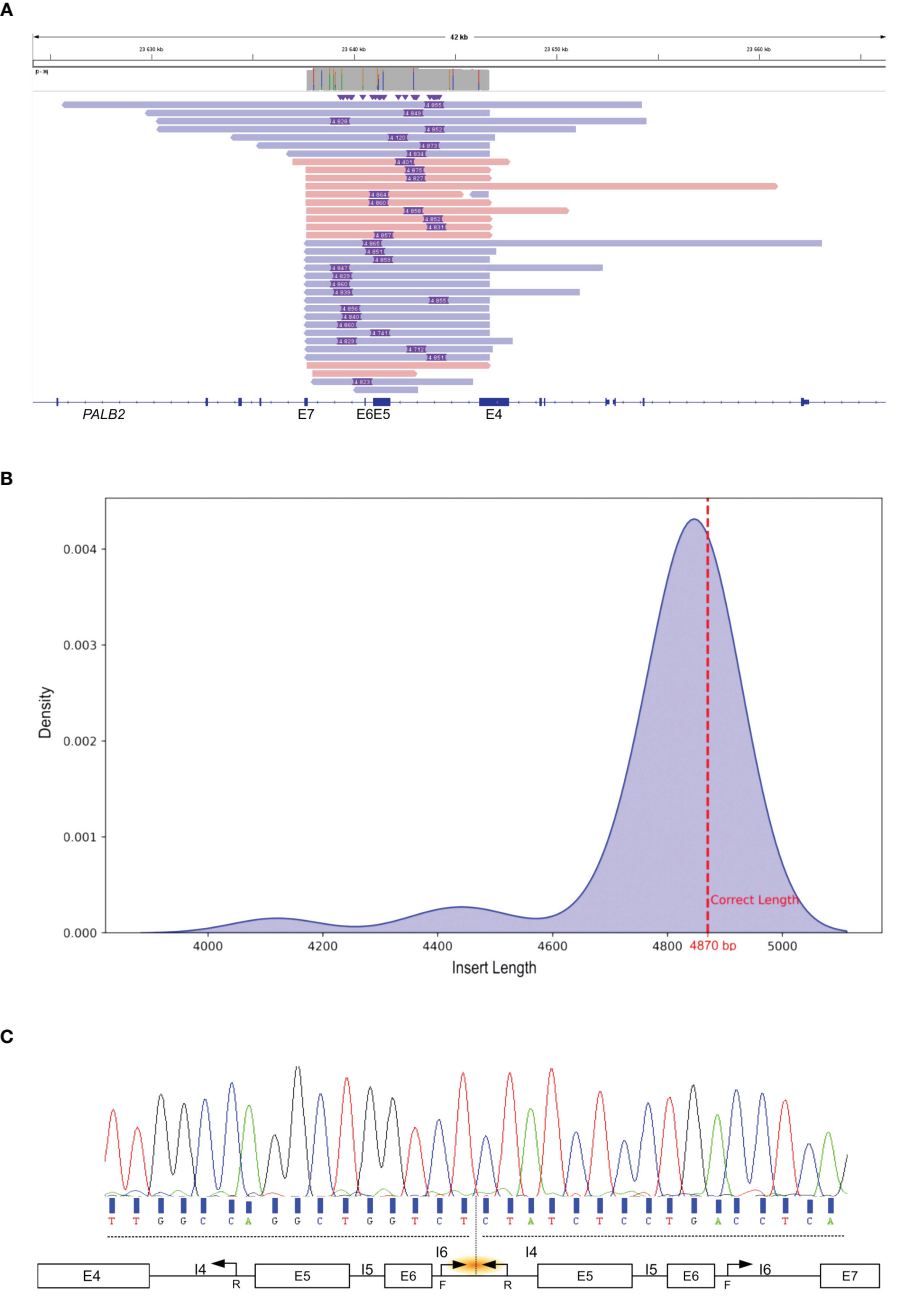
Identification and characterization of the Tandem Duplication Breakpoint in *PALB2*. **(A)** IGV visualization of Nanopore Long-Read Sequencing. Purple rectangle depict the genomic length of the insertion. The colored bars, in shades of red and blue, represent sequencing reads that span *PALB2* exons 4 to 7. **(B)** Density estimation plot of insertion length detected by long read sequencing. The discontinuous red line shows the exact insertion length determined by Sanger sequencing. **(C)** Sanger sequencing electropherogram of the genomic breakpoint region. Black arrows mark the primer positions. The orange shallow points the targeted region. F, forward; R, reverse; I, intron; E, exon.

To determine the genomic locations of the duplication breakpoints within *PALB2*, long-read sequencing was performed. After sequence alignment, this approach enabled the identification of insertions of different lengths (**Figure 2A**). Density estimation of the insertion length revealed an average insert size of duplication of 4,830 base pairs (**Figure 2B**). Local alignment of the inserted sequence revealed that the predicted proximal breakpoint originated within intron 4 of *PALB2* around genomic locus chr16:23644426 and the distal breakpoint within intron 6 around chr16:23639593. To confirm this observation and precisely map the breakpoints, we designed primers to amplify a region encompassing 200 bp upstream and downstream of the anticipated sites. Sanger sequencing confirmed the precise boundaries of the duplication with the exact coordinates identified as chr16: 23639343_23644213dup (NM_024675.4 *PALB2*: c.1684 + 1970_2586 + 1182dup) of 4,870 bp (**Figure 2C**).

Next, we examined breakpoint sequences to gain insight into the tandem duplication mechanism. Our analysis revealed a region of microhomology at both proximal and distal breakpoints with a 26 nucleotide sequence showing 100% identity. The nucleotide stretch was found within *Alu* elements: *AluSq2* in intron 4 and *AluSz6* in intron 6. The genomic sequences of the *Alu* element *AluSq2* and *AluSz6* had a homology of 74% (**Supplementary Figure 2**). The co-directional orientation of these *Alu* elements, in alignment with the transcriptional direction of *PALB2*, suggests a possible role of these repetitive elements in facilitating the duplication event.

## 4 Discussion

Our comprehensive genomic and transcriptomic analyses elucidated the pathogenic nature of an intragenic duplication involving exons 5 and 6 of *PALB2* gene in a patient with triple-negative breast cancer. The transition of this duplication from a class 3 VUS to a class 5 Pathogenic Variant (PV) aligns with the criteria set by the American College ofMedical Genetics and Genomics (ACMG). The supporting evidence for this reclassification is grounded in the demonstration of the duplication’s disruptive impact on the reading frame, resulting in a premature termination codon that is expected to lead to a truncated, dysfunctional protein p.(Asn863Glyfs*37). Moreover, the absence of a familial cancer history, combined with the presence of a *PALB2* intragenic duplication in the patient, raises the possibility of a *de novo* germline variant. Therefore, it is imperative to perform genetic testing on the immediate family, specifically the patient’s parents and children, to ascertain whether the duplication is inherited or arose spontaneously. This testing is crucial for accurate cancer risk assessment and underscores the importance of vigilant monitoring and preventive care within the family. In accordance with the most recent guidelines from the American College of Medical Genetics (ACMG) for individuals identified as carriers of genetic variants, it is recommended to implement enhanced surveillance strategies, including regular mammography and MRI screenings. These screenings should be personalized based on individual risk factors, such as age of onset in the family and personal medical history. Furthermore, owing to the moderate to high breast cancer risks associated with *PALB2* variants, it is advisable to discuss risk-reducing options, such as surgical preventive interventions.

The identification of *Alu* elements at both breakpoints provided a plausible mechanism for the origin of this duplication. The intra-strand slipped mispairing model, a form of non-allelic homologous recombination, is a recognized mechanism that gives rise to LGR (15). Our findings are consistent with this model, as the presence of homologous *Alu* sequences in co-directional orientation to the *PALB2* gene likely mediated misalignment during DNA replication or repair processes.

The pathological significance of this duplication is underscored by the aggressive clinicopathological features exhibited by the carrier, reflecting a phenotype that other studies have associated with loss-of-function variants of *PALB2* (9, 10, 16). This connection reinforces the role of *PALB2* as a critical player in DNA damage repair through the homologous recombination pathway. Furthermore, the observation of microhomology at the duplication breakpoints is in line with recent studies indicating that such regions are hotspots for genomic instability (13), thereby contributing to the pathogenicity of LGR.

As our work, Kwong and colleagues also highlight the usefulness of long-read sequencing technologies, such as Nanopore, for the in-depth examination of LGRs [*Ava Kwong et al, JCO Precis Oncol 5, 2021*]. Specifically, when LGR impact critical gene regions, such as the first or last exons, conventional short-read NGS often encounters challenges, particularly in accurately resolving low-complexity stretches and determining homopolymer lengths, factors critical for accurately pinpointing breakpoints, orientations, and positioning within the genome. Thus, PCR-free Nanopore sequencing, as demonstrated by its ability to characterize complex LGR, offers a cost-effective and efficient alternative to more traditional and labor-intensive methods. However, this long-read technology is not suitable for clinical tumor samples, as sequencing gDNA extracted from formalin-fixed, paraffin-embedded (FFPE) samples is highly challenging due to DNA degradation and the limited amounts of sample available.

In conclusion, our findings not only broaden the range of known pathogenic variants linked to HBOC syndrome but also highlight long-read sequencing as a possible and faster tool for detailed characterization of structural variations. This heightened diagnostic accuracy promises to enhance risk assessment and management strategies as well as therapeutic interventions for hereditary cancer syndromes.

## Data availability statement

The study's original contributions are included in the article and **Supplementary Material**. Further inquiries can be directed to the corresponding author.

## Ethics statement

The studies involving humans were approved by Montpellier University Hospital. The studies were conducted in accordance with the local legislation and institutional requirements. The participants provided their written informed consent to participate in this study. Written informed consent was obtained from the individual(s) for the publication of any potentially identifiable images or data included in this article.

## Author contributions

IB: Conceptualization, Data curation, Formal analysis, Investigation, Methodology, Software, Supervision, Validation, Visualization, Writing – original draft, Writing – review & editing. AC: Investigation, Resources, Writing – review & editing, Software. TG: Data curation, Software, Writing – review & editing. JP: Formal analysis, Writing – review & editing. SC-A: Visualization, Writing – review & editing, Software. PP: Resources, Writing – review & editing. JV: Visualization, Writing – review & editing, Data curation, Formal analysis, Methodology, Software, Validation. JS: Project administration, Supervision, Validation, Writing – review & editing, Funding acquisition, Methodology, Resources, Visualization.

## Supplementary material

SUPPLEMENTARY FIGURE 1 Genealogy tree. The pedigree presents a four generation lineage, with the proband marked by an arrow. Black-filled circle represent the family member affected by cancer, with the age at diagnosis noted underneath.

SUPPLEMENTARY FIGURE 2 Sequence Alignment of the *AluSq2* and *AluSz6* elements involved in the *PALB2* exons 5 and 6 duplication. The image shows a comparison of the two Alu sequences, matching nucleotides between the sequences are marked with an asterisk (*). The numbers on the right indicate the nucleotide positions in the sequence alignment.
