# Supplementary figures and images for: Characterizing PALB2 intragenic duplication breakpoints in a triple-negative breast cancer case using long-read sequencing
Source: Front Oncol. 2024 Feb 28;14:1355715. doi: 10.3389/fonc.2024.1355715 (PMC10938850; doi:10.3389/fonc.2024.1355715)

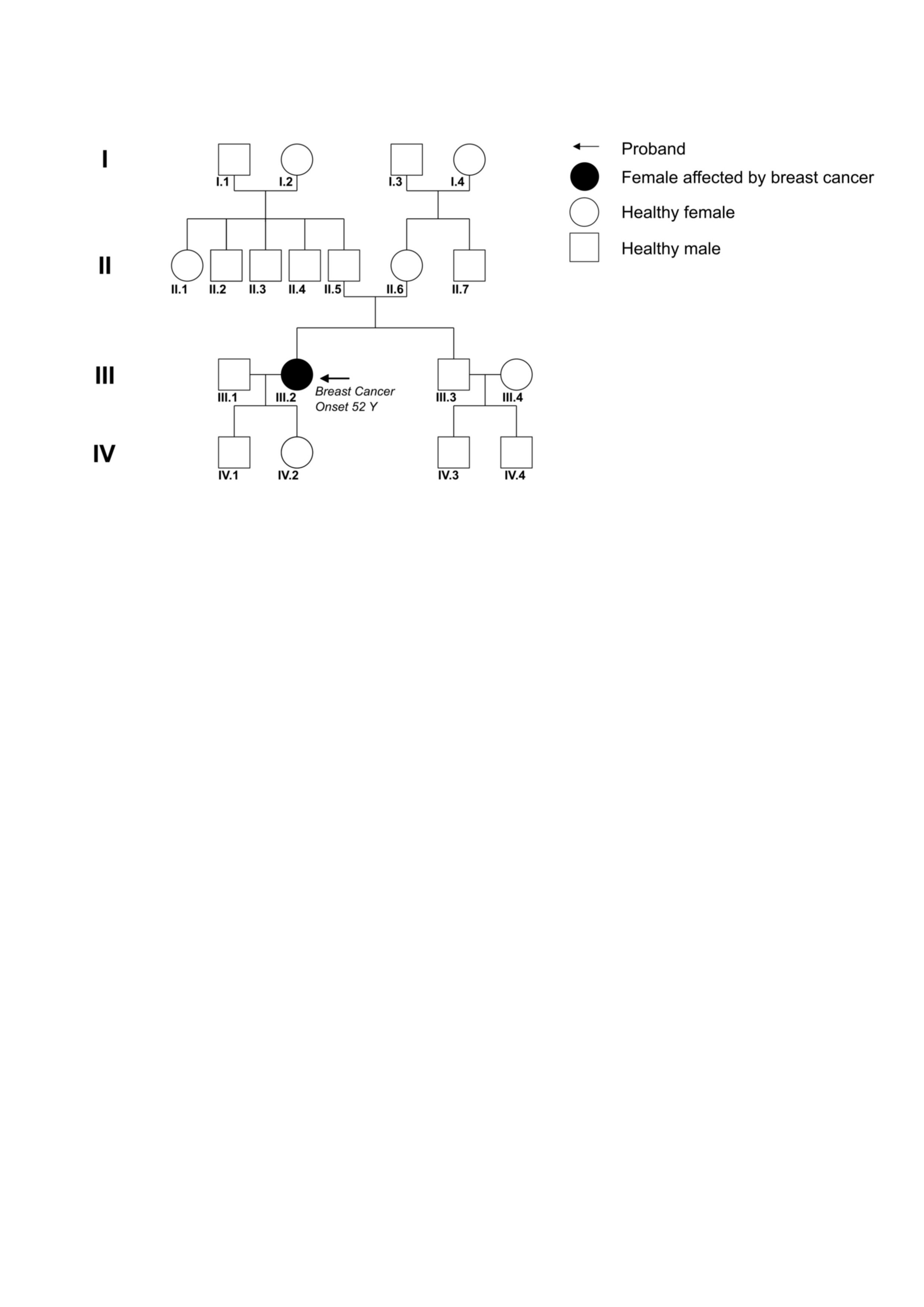

Supplement: SUPPLEMENTARY FIGURE 1 — Genealogy tree. The pedigree presents a four generation lineage, with the proband marked by an arrow. Black-filled circle represent the family member affected by cancer, with the age at diagnosis noted underneath. [file Image_1.jpg]

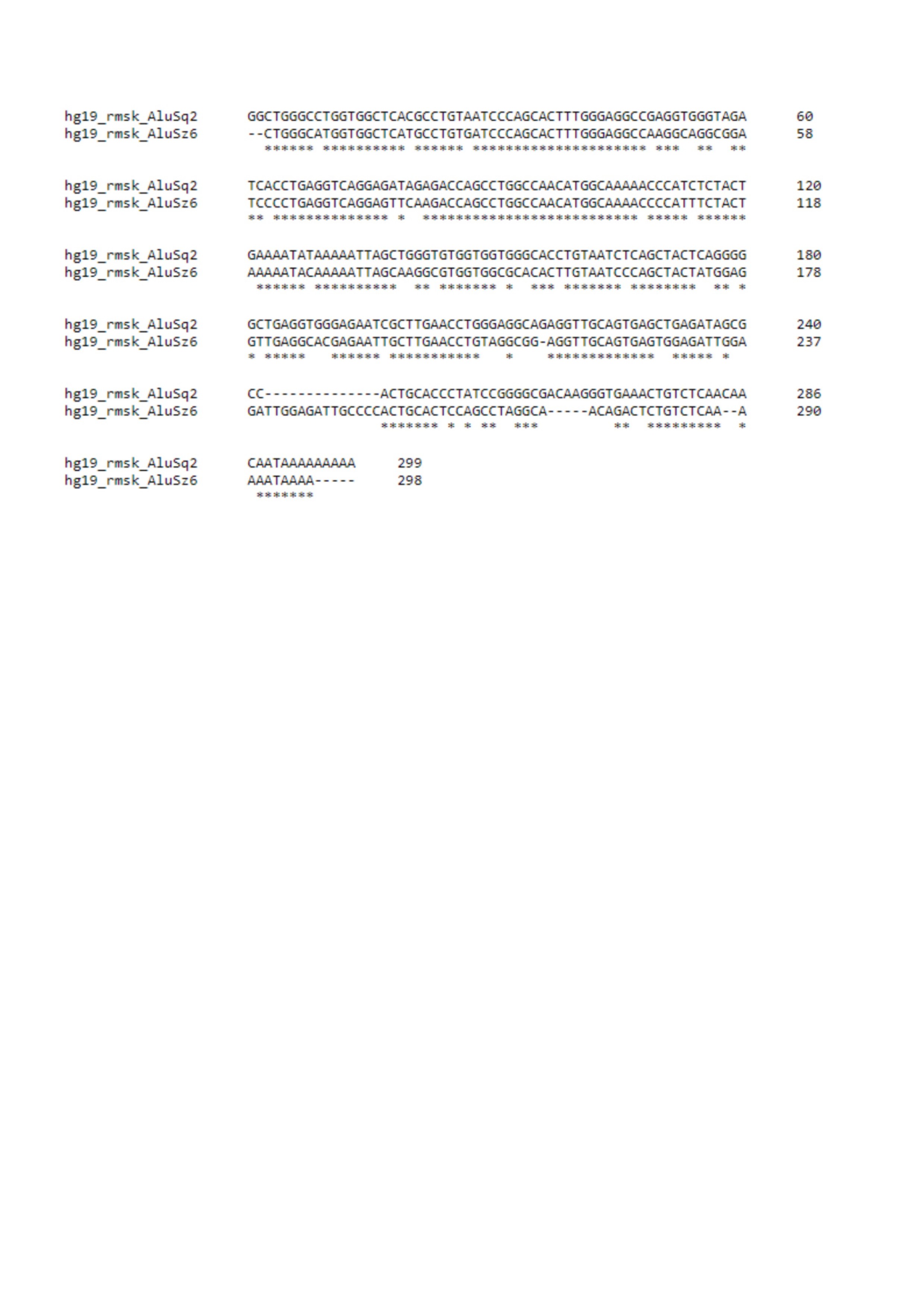

Supplement: SUPPLEMENTARY FIGURE 2 — Sequence Alignment of the AluSq2 and AluSz6 elements involved in the PALB2 exons 5 and 6 duplication. The image shows a comparison of the two Alu sequences, matching nucleotides between the sequences are marked with an asterisk (*). The numbers on the right indicate the nucleotide positions in the sequence alignment. [file Image_2.jpg]
